# Association of the microsatellite in the 3' untranslated region of the *CD154 *gene with rheumatoid arthritis in females from a Spanish cohort: a case-control study

**DOI:** 10.1186/ar2288

**Published:** 2007-09-10

**Authors:** Trinidad Martin-Donaire, Ignacio Losada-Fernandez, Gema Perez-Chacon, Iñigo Rua-Figueroa, Celia Erausquin, Antonio Naranjo-Hernandez, Silvia Rosado, Florentino Sanchez, Ayoze Garcia-Saavedra, Maria Jesus Citores, Juan A Vargas, Paloma Perez-Aciego

**Affiliations:** 1 Fundacion LAIR, Madrid, Spain; 2 Servicio de Medicina Interna I, Hospital Universitario Puerta de Hierro, Universidad Autonoma de Madrid, C/San Martin de Porres 4, 28035 Madrid, Spain; 3 Servicio de Reumatologia, Hospital Universitario de Gran Canaria Doctor Negrin, Barranco de la Ballena s/n, 35010 Las Palmas de Gran Canaria, Spain; 4 Servicio de Inmunologia, Hospital Universitario de Gran Canaria Doctor Negrin, Barranco de la Ballena s/n, 35010 Las Palmas de Gran Canaria, Spain

## Abstract

CD40–CD154 interaction is an important mediator of inflammation and has been implicated in T helper type 1-mediated autoimmune diseases including rheumatoid arthritis (RA). Linkage studies have shown association of markers in the proximity of the *CD154 *gene. In the present work we investigated whether specific allele variants of the microsatellite in the 3' UTR of the *CD154 *gene might modulate the risk of RA. The study, in a case-control setting, included 189 patients and 150 healthy controls from the Canary Islands, Spain. The *24CAs *allele was less represented in female patients than in controls (0.444 in controls versus 0.307 in patients, *P *= 0.006, odds ratio (OR) 0.556, 95% confidence interval (CI) 0.372 to 0.831) but not in males (0.414 versus 0.408), and only when homozygous (*P *= 0.012; OR 0.35, 95% CI 0.16 to 0.77). We also verified that CD154 association with RA was independent of human leukocyte antigen (HLA) phenotype. A further functional study showed that after stimulation anti-CD3, CD154 mRNA was more stable in CD4^+ ^T lymphocytes from patients with RA bearing the *24CAs *allele (mRNA half-life 208 minutes) than in patients without the *24CAs *allele (109 minutes, *P *= 0.009). However, a lower percentage of CD154^+^CD4^+ ^T lymphocytes was seen in freshly isolated peripheral blood mononuclear cells from patients carrying *24CAs *alleles (mean 4.28 versus 8.12; *P *= 0.033), and also in CD4^+ ^T lymphocytes stimulated with anti-CD3 (median 29.40 versus 47.60; *P *= 0.025). These results were concordant with the smaller amounts of CD154 mRNA isolated from stimulated T lymphocytes with *24CAs *alleles. The *CD154 *microsatellite therefore seems to affect the expression of the gene in a complex manner that implies not only mRNA stability. These data suggest that the *CD154 *microsatellite contributes to the regulation of mRNA and protein expression, although further studies will be necessary to elucidate its role in disease predisposition.

## Introduction

Rheumatoid arthritis (RA) is a chronic relapsing inflammatory condition of unknown etiology [1]. The disease is characterized by persistent inflammatory synovitis leading to joint destruction and is sometimes associated with systemic involvement [2]. Clinical expression of the disease ranges from a mild, non-deforming arthropathy with little long-term disability, to severe, incapacitating, deforming arthritis, which may be refractory to conventional disease-modifying agents [3]. Because early prescription of a disease-modifying anti-rheumatic drug may be more effective in controlling severe disease, early diagnosis and prediction of severity are important [4,5]. The identification of markers associated with susceptibility to or severity of RA is therefore currently an important task.

Twin and family studies provide evidence to support the involvement of both genetic and environmental factors in the etiopathogenesis of RA [6,7]. Epidemiological studies show an important genetic background in RA, with the major histocompatibility complex (MHC) region showing the strongest association with disease predisposition, although the contribution of the human leukocyte antigen (HLA) genes has been estimated to be no more than 30 to 50% to the total genetic background [8]. Thus, several other genes outside the MHC locus are likely to be involved, probably each contributing a small amount to the genetic predisposition to RA [7]. Recent findings from linkage studies have drawn attention to several regions that probably contain candidate genes, namely 1p, 5q, 8p, 12, 13, 18q, 21q and the X chromosome [9-15], for which association studies are needed.

It is believed that the pathology and etiology of RA involve abnormal presentation of self antigen(s) by antigen-presenting cells (APCs) and the activation of autoreactive T cells [16]. Several costimulatory molecules are involved during interactions between APCs and T cells, namely CD40 and CD40 ligand (CD154), which are required for the amplification of the inflammatory response [16]. In RA, T cells expressing CD40 ligand infiltrate the synovial fluid and interact with fibroblasts expressing CD40, which induces fibroblast proliferation [17], increased recruitment of inflammatory cells [18], and the production of tumor necrosis factor-α [19]. In addition, the production of IL-12 by synovial fluid macrophages, which is required for the initiation of T helper type 1 (Th1) cell responses, is regulated by the CD40–CD154 interaction [20]. Because RA is mediated by Th1 cells, CD40–CD154 interaction may be an important pathogenic pathway [21]. Indeed, CD4^+ ^T cells from patients with RA have an increased expression of CD154 [21-24] that is still observed 5 to 12 years after disease onset, indicating augmented and prolonged activation of T cells.

The *CD154 *gene is located on the X chromosome and belongs to the tumor necrosis factor gene family [25]. It contains a dinucleotide repeat of cytosine-adenine (CA) in the 3' UTR that because of its location may have some bearing on the regulation of gene expression. Although CD154 is regulated both temporally and with respect to the cell type, the underlying mechanisms responsible for this control have not yet been completely elucidated. *CD154 *gene transcription is induced by TCR signaling and expression is enhanced in response to costimulatory signals. Transcriptional regulation seems to be dependent on NF-AT and NF-κB binding sites located in the promoter region [26]. Binding sites for AP-1 and a CD28 response element have also been described [27], and a NF-κB binding site with enhancer activity has been found downstream of the poly(A) signal site [28]. In addition to transcriptional regulation, it has been shown that post-transcriptional regulation also has a crucial role in modulating the expression of the *CD154 *gene. As with other cytokine genes, the 3' UTR of the *CD154 *mRNA contains binding sites for RNA–protein complexes that are responsible for the lability of the mRNA. It has been found that the mRNA decay rate can be specifically modified in some situations, and the protein complexes involved in this regulation are being characterized [29-31]. It has been proposed that a putative stability complex binds specifically to a highly pyrimidine-rich region in the 3' UTR, and this complex seems to be directly involved in regulating the variable decay rate of *CD154 *mRNA during T cell activation [32].

Allele distribution for the dinucleotide-repeat polymorphism located in the 3' UTR of the *CD154 *gene has previously been investigated [33,34]. Allele variants of this polymorphism have been found to be associated with RA in a subgroup of German patients [33] and with systemic lupus erythematosus in Spaniards [35] but not with multiple sclerosis in Nordic patients [36]. The aim of the present work was to study whether specific allele variants of this gene might modulate the risk of RA in Spaniards from the Canary Islands. We also investigated the influence of the allele variants of *CD154 *on mRNA and protein expression in peripheral-blood T cells from patients with RA.

## Materials and methods

### Patients and controls

The study used a case-control design to compare patients and controls. A total of 189 patients diagnosed with RA according to the American College of Rheumatology criteria were enrolled at the Rheumatology Unit at the Dr Negrin General Hospital from Gran Canaria (Canary Islands, Spain). The median age at onset of RA was 45 years (interquartile range 27 to 63 years) and the median disease duration was 13 years (interquartile range 2 to 24); 74% of the patients with RA were female, 78% were positive for rheumatoid factor, 80% had demonstrated erosions, and 31% presented extra-articular manifestations. All had received antimalarials or disease-modifying anti-rheumatic drugs. Control subjects (150 in all; namely 70 males and 80 females) matched by age and geography and with no history of inflammatory arthritis were recruited. All participants gave their written informed consent.

### Samples

Peripheral blood was obtained from patients and controls, and genomic DNA was extracted by digestion with proteinase K and extraction with phenol/chloroform [37] (Sigma-Aldrich, St Louis, MO, USA). Peripheral blood mononuclear cells (PBMCs) were obtained by density gradient centrifugation with Lymphocytes Isolation Solution (Comercial Rafer SL, Zaragoza, Spain).

### *CD154 *microsatellite typing

A segment of the 3' UTR of the *CD154 *gene containing the microsatellite was amplified by PCR, and the amplification products were resolved over denaturing polyacrylamide gels as described previously [34]. Allele assignment was performed by densitometry with the Quantity One^® ^Software (Bio-Rad Laboratories, Hercules, CA, USA). The length of the amplified fragment was estimated by reference to the standards used as internal ladder, and the number of repeats was calculated from the published sequence (GenBank accession number D31797). In 10 samples genotype assignments were confirmed with an ABIPRISM 3730 system (Applied Biosystems, Foster City, CA, USA).

### HLA typing

HLA class II (*DRB1 *and *DQB1*) alleles were studied by PCR and sequence-specific oligonucleotides hybridization (PCR-SSO) using LIFEMATCH™ HLA-SSO DNA Typing kits (Orchid Diagnostics, Stamford, CT, USA), in accordance with the manufacturer's instructions.

### Cell cultures

PBMCs were cultured at 37°C in a humidified 5% CO_2 _atmosphere in RPMI 1640 medium supplemented with 10% heat-inactivated fetal bovine serum, 100 units/ml penicillin, 100 μg/ml streptomycin and 2 mM L-glutamine (all from Gibco, Life Technologies Inc., Rockville, MD, USA). T lymphocytes were expanded *in vitro *by culturing PBMCs at 2 × 10^5 ^cells/ml in six-well culture plates (Costar, Cambridge, MA, USA) with 5 μg/ml phytohemagglutinin (PHA; Difco Laboratories, Detroit, MI, USA), 62.5 ng/ml anti-CD28 soluble mAb (Kolt-2; Menarini, Badalona, Spain), and 50 units/ml recombinant human IL-2 (Proleukin^®^; Chiron BV, Amsterdam, Holland). After a week, more than 95% of the cells in the culture were CD3^+ ^resting T lymphocytes, as confirmed by flow cytometry.

For stimulation of the PBMCs or expanded T cells with anti-CD3 mAb, 24-well culture plates (Costar) were coated overnight at 4°C with 50 μg/ml anti-CD3 mAb (Orthoclone OKT^®^3; Cilag AG Int., Zug, Switzerland) in 50 mM Tris-HCl pH 9.5. After incubation overnight, coating solutions were removed and plates were washed gently with RPMI 1640 medium to remove unbound mAb. Cells were cultured at 5 × 10^5 ^cells/ml on anti-CD3 coated plates with 62.5 ng/ml anti-CD28 soluble mAb for 6, 24, 48, 72, or 92 hours, depending on the assay.

### mRNA decay assays

Expanded T cells, once they were resting, were restimulated with anti-CD3 plus anti-CD28 for 6 or 24 hours as mentioned above. Then, 10 μg/ml actinomycin D (ActD; Sigma-Aldrich), a transcriptional inhibitor, was added to the culture and aliquots of cells were collected at different time points for RNA extraction. Total RNA was isolated by the guanidinium thiocyanate method by using the Trizol reagent (Gibco) [38] and transcribed to cDNA with AMV reverse transcriptase (Roche Diagnostics, Gmbh, Mannheim, Germany), in accordance with the manufacturer's instructions. *CD154 *mRNA was measured by using a quantitative competitive PCR kit for human CD154 (Maxim Bio, San Francisco, CA, USA), in accordance with the manufacturer's instructions. Then, 10 μl of each reaction was subjected to electrophoresis on 2% NuSieve 3:1 agarose gels (Cambrex Bio Science Rockland, Rockland, MA, USA), and revealed by staining with ethidium bromide (Sigma-Aldrich). PCR products were quantified by using the Quantity One Software with reference to the standard from the kit. In this technique, serial dilutions of known quantities of PCR competitor are added to PCR reactions containing a constant amount of target cDNA. The molar ratio of PCR and competitor remains constant during the reaction, so the initial amount of target cDNA molecules can be calculated from the known number of competitor molecules added to the reaction as [(moles of target CD154 RNA) × (6 × 10^23 ^molecules per mole) × (dilution factor of test RNA)]/(μg of total RNA). The number of CD154 mRNA molecules, quantified as indicated above, was then corrected for the proportion of CD4^+ ^cells in each sample and expressed as molecules per μg of total RNA in CD4^+ ^T lymphocytes, assuming equal RNA content between T cell subtypes. For the determination of mRNA half-lives (*t*_1/2_), fractions of *CD154 *mRNA remaining after the addition of ActD were plotted against time after ActD addition. After exponential adjustment of curves, mRNA half-lives were calculated as the time in which the fraction of mRNA remaining decreased to 50% of the initial amount.

### CD154 surface expression

Freshly isolated PBMCs or stimulated T cell suspensions were washed and stained with anti-human CD45, CD3, CD14, CD4, CD69, CD25, and CD154 mAbs (all from BD Biosciences, San Jose, CA, USA). Labeled cells were then analyzed in a FACSort flow cytometer with the CellQuest^® ^software (BD Immunocytometry Systems, San Jose, CA, USA). The percentage of CD154-positive cells was calculated by subtracting overlaid CD154 and isotype control (Ig) histograms. Mean fluorescence intensity (MFI) was quantified on a linear scale as the ratio of the geometric mean of the CD154-phycoerythrin antibodies against the irrelevant anti-mouse-IgG-phycoerythrin antibodies of total CD4^+ ^T cells.

### Apoptosis assays

Apoptotic cells in culture were detected by staining with fluorescein isothiocyanate (FITC)-labeled annexin-V (Roche Diagnostics) and propidium iodide (Sigma-Aldrich). After 15 minutes in the dark, cells were analyzed by flow cytometry. Cell viability was measured as the percentage of cells that were negative for both annexin-V and propidium iodide.

### Proliferation assays

PBMCs were stimulated with anti-CD3 plus anti-CD28 for three days, subsequently pulsed with 60 μM bromodeoxyuridine (BrdU) (Sigma-Aldrich), and harvested 18 hours later. The incorporation of BrdU was measured by staining with an FITC-conjugated anti-BrdU antibody (BD Biosciences) and analyzed by flow cytometry.

### Statistical analysis

Allele and genotype frequencies and carrier rates were calculated in patients with RA and in controls, and no deviations from Hardy–Weinberg equilibrium in controls were confirmed by comparison of observed and expected genotype frequencies [39]. Differences in allele/genotype frequencies between patients and healthy control subjects were tested by the χ^2 ^method, using the Yates or Bonferroni correction or Fisher's exact test when appropriate. The strength of association between RA and alleles of *CD154*, *DRB1 *and *DQB1 *was estimated by using odds ratios (ORs) and the exact limits of the 95% confidence intervals (CIs). Estimation of the statistical power for the comparison of allele frequencies was performed with the STPLAN software. The arcsin approximation of the binomial distributions of allele frequencies was used with a two-sided test and with α fixed at 0.05. To examine interactions between variables associated with RA we conducted a multivariate analysis with a binary logistic regression model. Surface expression levels of *CD154 *mRNA and CD154 protein were compared by using the non-parametric Mann–Whitney test. The statistical package SSPS for Windows v. 10 (SSPS Inc., Chicago, IL, USA) was used. *P *< 0.05 was considered statistically significant.

## Results

### *CD154 *microsatellite is associated with RA in females

Overall, allele frequencies (Additional file 1) did not differ between patients and controls after applying the Bonferroni correction to the χ^2 ^test (*p*_c _= 0.34). However, comparison of the frequencies of each allele between patients and controls showed differences for the *24CAs *allele (0.32 versus 0.44; *P *= 0.009; OR 0.62, 95% CI 0.44 to 0.88; power 0.70) and the *26CAs *allele (0.088 versus 0.030; *P *= 0.014; OR 2.96, 95% CI 1.27 to 6.91; power 0.80). Because *CD154 *is located on the X chromosome, we compared allele frequencies between patients and controls in males and females separately. We observed statistical differences in the *24CAs *allele frequency in females (0.44 in healthy controls versus 0.31 in patients; *P *= 0.006; OR 0.56, 95% CI 0.37 to 0.83; power 0.82) but not in males (0.41 versus 0.41). Similarly, differences were found in the *26CAs *allele in females (0.03 in healthy controls versus 0.09 in patients; *P *= 0.033; OR 3.04, 95% CI 1.14 to 8.10; power 0.78) but not in males (0.03 versus 0.06). These data suggested a possible disease-protective role for the *24CAs *variant in females. However, for the *26CAs *allele, its contribution to disease predisposition does not seem to be relevant because of the low incidence in both patients and controls.

**Figure 1:**
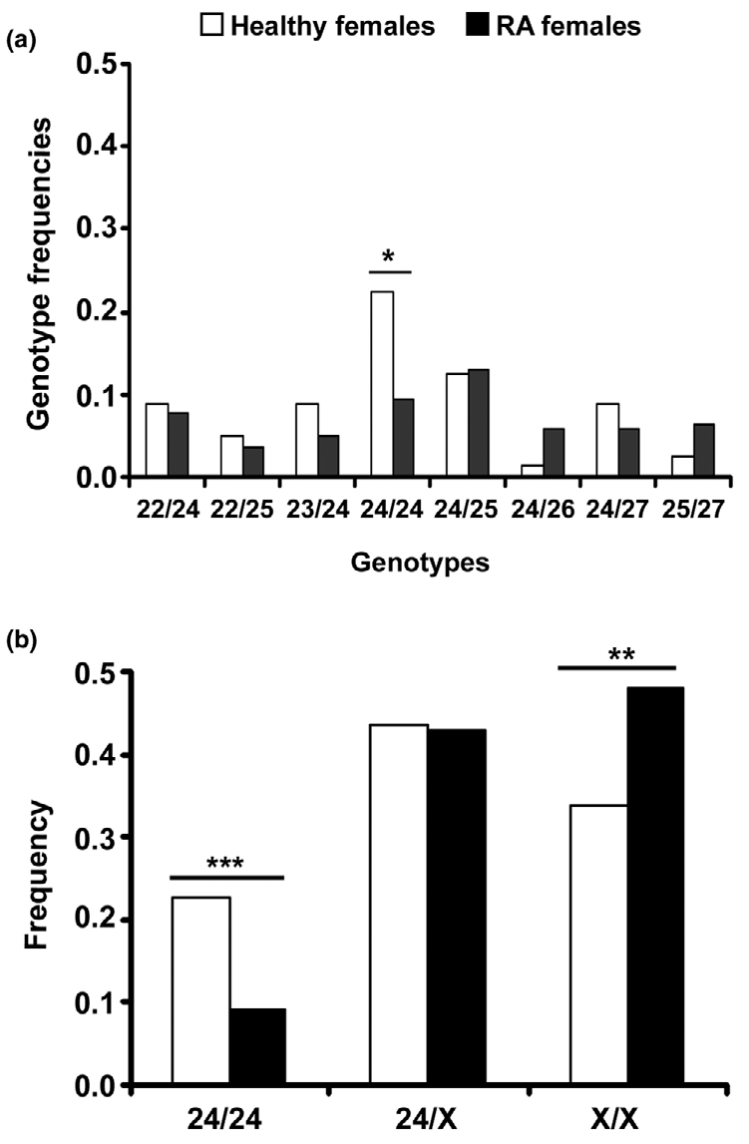
Genotype frequencies of the *CD154 *microsatellite in healthy females and those with rheumatoid arthritis. **(a) **Seventy-nine female patients with rheumatoid arthritis (RA) were compared with 56 healthy females (*P*_c _= 0.483). Genotypes represented fewer than five times are not included. *P = 0.012. **(b) **The frequency for carriers of two (24/24), one (24/X) or zero (X/X) alleles of *24CAs*, where X represents any allele different from *24CAs*. One hundred and forty female patients with RA were compared with 80 healthy females (*P*_c _= 0.026). **P = 0.042, ***P = 0.012.

Next, we studied genotype frequencies in females, and we found a lower frequency of the *24CAs*/*24CAs *homozygous genotype (*P *= 0.012; OR 0.35, 95% CI 0.16 to 0.77) in patients with RA than in healthy controls (Figure 1a). We then classified females as being carriers of two (24/24), one (24/X) or zero (X/X) *24CAs *alleles by comparing these genotype frequencies. As can be seen in Figure 1b, RA females bearing *24CAs*/*24CAs *were less frequent (*P *= 0.012), whereas X/X cases were more frequent (*P *= 0.042) than in healthy controls, indicating that the *24CAs *allele seems to protect from RA when homozygous.

### Association of *CD154 *with RA is independent of *HLA-DRB1 *and *HLA-DQB1*

Epidemiological studies show a strong association of MHC region with disease predisposition, being related to the presence of 'shared epitopes' of *HLA-DRB1*, which includes the DRB1*04 and DRB1*01 alleles. To confirm whether the observed '*CD154 *association' could be influenced by HLA phenotype, we typed *DRB1 *and *DQB1 *genes in our patients and controls by low-resolution PCR-SSO techniques. Patients with RA from the Canary Islands showed higher allele frequencies of DR4 (0.27 versus 0.12 in controls; *P *< 0.0005; OR 2.68, 95% CI 1.65 to 4.35) and DQ3 (0.39 versus 0.27 in controls; *P *= 0.005; OR 1.74, 95% CI 1.20 to 2.54), confirming the association of these variants with RA previously described in Spaniards [40,41].

Next, we analyzed the distribution of pairs of variables in patient and control groups in contingency tables to test whether the association of any variable with RA depended on the presence of any other variable. This analysis revealed that the association of DQ3 with RA was dependent on DR4, as expected from the linkage of both genes (data not shown) and that *CD154 *was associated with RA independently of the presence of DR4 (Table 1). The influence of sex in the associated variables was analyzed by using a multivariate binary logistic regression model. The final model included DR4, *24CAs *and sex as independent variables, and disease as the dependent variable. As can be seen in Table 2, the association of *CD154-24CAs*, but not DR4, with RA is affected by sex.

**Table 1:** Distribution of *24CAs *carriers among DR4^+ ^and DR4^- ^patients with RA and healthy controls

Allele	DR4^-^			DR4^+^		
	RA (*n *= 98)	Healthy controls (*n *= 77)	*P*	RA (*n *= 91)	Healthy controls (*n *= 22)	*P*
Non-*24CAs*	51 (52)	31 (40.3)	0.121	45 (49.5)	11 (50)	0.963
*24CAs*	47 (48)	46 (50.5)		46 (59.7)	11 (50)	

*n*, number of samples analyzed. Results in parentheses are percentages.

**Table 2:** Binary logistic regression model showing influence of sex on *CD154 *gene association with RA

Parameter	Coefficient	Standard error	OR (95% CI)	*P*^a^
Constant	-0.242	0.227		
DR4	1.109	0.289	3.03 (1.72–5.34)	<0.001
Sex	1.386	0.368	4.00 (1.95–8.22)	<0.001
*CD154-24CAs *by sex	-0.946	0.362	0.39 (0.19–0.79)	0.009

OR, odds ratio; CI, confidence interval. ^a^*p *value based on Wald statistic.

### *CD154 *microsatellite influences mRNA stability in T lymphocytes

Because of the proximity of the *CD154 *microsatellite to sites regulating mRNA stability [30,32], we considered studying whether this polymorphism could affect the *CD154 *mRNA half-life. This gene is located on the X chromosome, so we selected homozygotic patients with RA to assign the phenotype to a single allele; these individuals were then stratified by genotype. It is known that activation of peripheral T lymphocytes in patients with RA can fluctuate, affecting to the degree of apoptosis or response to mitogens *in vitro*. To avoid this heterogeneity, we first used PHA, anti-CD28 and IL-2 to stimulate PBMCs from 20 patients. After 1 week in culture, homogeneous cellular populations were obtained with more than 95% of resting CD3^+ ^lymphocytes, as confirmed by CD69 staining. The CD4/CD8 ratio of these cells did not differ between *24CAs *and non-*24CAs *patients (2.02 and 1.82, respectively; *P *= 0.037). Anti-CD3 stimulation for 24 hours or more has been shown to specifically stabilize the normally unstable *CD154 *mRNA, augmenting its half-life notably [29]. We therefore stimulated the previously expanded T cells with anti-CD3 and anti-CD28 for 6 or 24 hours to analyze the effect of the microsatellite in both situations. After that, cells were treated with the transcriptional inhibitor ActD, and the decay of *CD154 *mRNA was determined by quantitative competitive reverse transcriptase-mediated PCR.

**Figure 2:**
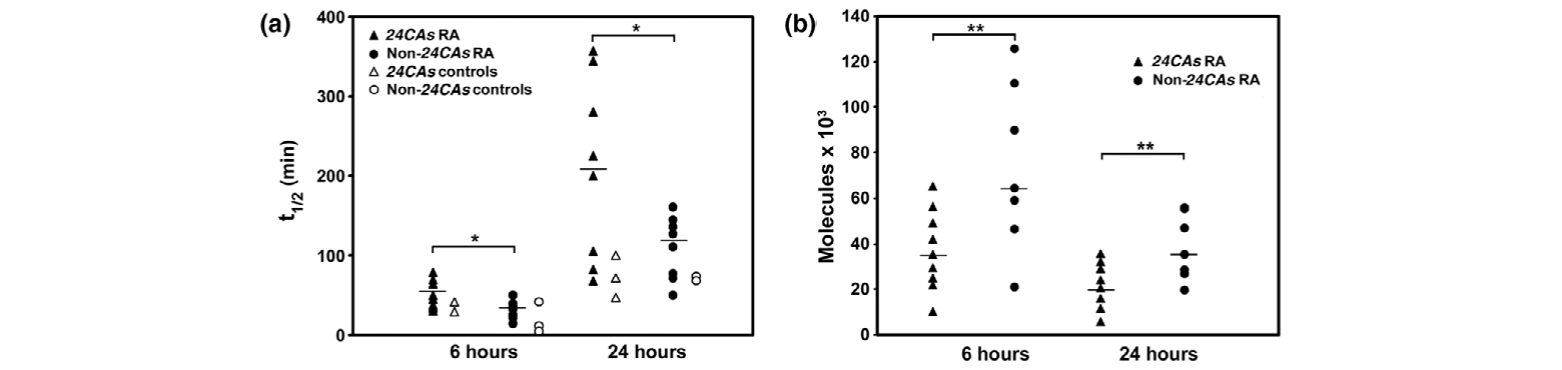
Expression of *CD154 *mRNA in T lymphocytes according to *CD154 *genotype. T lymphocytes from patients with rheumatoid arthritis (RA; *24CAs *group, *n *= 9; non-*24CAs *group, *n *= 11) and controls (*24CAs *group, *n *= 3; non-*24CAs *group, *n *= 3), obtained after *in vitro *expansion of peripheral blood mononuclear cells, were stimulated for 6 or 24 hours with anti-CD3 plus anti-CD28; after this, mRNA decay assays were performed as indicated in the Materials and methods section. **(a) **mRNA half-life, *t*_1/2_, in T lymphocytes. **(b) **mRNA molecules per μg of total RNA in CD4^+ ^T lymphocytes. *P < 0.05, **P < 0.005, comparing the *24CAs *group with the non-*24CAs *group.

After 6 hours of stimulation, we found statistically significant differences in the half-life of *CD154 *mRNA between *24CAs *(*t*_1/2 _49.7 ± 17.4 minutes (mean ± SD)) and non-*24CAs *alleles (32.2 ± 10.8 minutes; *P *= 0.005) (Figure 2a). After 24 hours of stimulation, there was a clear mRNA stabilization in both groups of patients (fourfold increase in the *24CAs *mRNA *t*_1/2 _(208 ± 115 minutes) in comparison with a threefold increase in non-*24CAs *mRNA *t*_1/2 _(109 ± 39 minutes); *P *= 0.009) (Figure 2a). In contrast with what we expected, the half-life of the *24CAs *mRNAs was higher than that of the rest of the alleles after 6 and 24 hours of anti-CD3 stimulation. As shown in Figure 2a, scattering of the data in the non-*24CAs *group was similar to that in the *24CAs *group. This indicated that the functional behavior of the samples included in the non-*24CAs *group, in spite of the inclusion of several different alleles, was as homogeneous as that in the *24CAs *samples, validating our stratification of patients by *24CAs *allele. A similar pattern was also observed in healthy individuals, although a statistical comparison was not performed for healthy samples because of the low sample numbers (Figure 2a).

However, if the number of molecules was compared instead of mRNA decay, the total number of *CD154 *mRNA molecules in CD4 T lymphocytes after 6 hours of stimulation with anti-CD3 plus anti-CD28 was significantly lower in patients with *24CAs *(6.181 ± 2.908 molecules (mean ± SD) of *CD154 *mRNA per μg of total RNA in CD4 T lymphocytes) than in those with non-*24CAs *alleles (21.254 ± 19.994; *P *< 0.05). The same occurred after 24 hours of stimulation with anti-CD3 plus anti-CD28 (3.461 ± 2.277 molecules of *CD154 *mRNA per μg of total RNA in CD4 T lymphocytes, versus 7.009 ± 8.637 in non-*24CAs*; *P *< 0.05; Figure 2b). In both cases these initial differences were shortened along the time in culture after the addition of ActD (probably as a result of the greater stability of the *24CAs *mRNA).

### *CD154 *microsatellite influences surface protein expression in T lymphocytes

We next tried to assess whether the *CD154 *microsatellite could affect protein expression in RA T lymphocytes, as we had previously described in healthy donors [35]. First, we compared the expression of several surface markers in freshly isolated PBMCs from patients with RA who were homozygous for *24CAs *or non-*24CAs *alleles. As shown in Table 3, patients with *24CAs *alleles displayed a higher percentage of CD19^+ ^B lymphocytes (*P *= 0.018) and of activated CD25^+^CD4^+ ^T lymphocytes (*P *= 0.036) but, surprisingly, a lower percentage of CD154^+^CD4^+ ^T lymphocytes (*P *= 0.033). The MFI was also higher in patients with RA who were homozygous for non-*24CAs *alleles (4.44 ± 1.02 versus 3.22 ± 1.10), although differences did not reach statistical significance (data not shown).

**Table 3:** Basal expression of surface markers in PBMCs of patients with RA according to *CD154 *genotype

Surface markers	*24CAs *(*n *= 13)	Non-*24CAs *(*n *= 15)	*P*
CD16/CD56^a^	16.07 ± 10.78	12.38 ± 10.24	n.s.
CD19^a^	6.42 ± 3.12	4.17 ± 3.01	0.018
CD3/CD8^a^	17.24 ± 7.66	22.37 ± 6.67	n.s.
CD3/CD4^a^	44.40 ± 14.79	41.22 ± 13.11	n.s.
CD4/CD69^b^	14.09 ± 19.02	7.48 ± 10.25	n.s.
CD4/CD25^b^	59.59 ± 18.04	41.19 ± 22.84	0.036
CD4/CD154^b^	4.28 ± 3.81	8.12 ± 5.73	0.033

Results are means ± SD for cells expressing the indicated surface markers, quantified by flow cytometry. Differences were evaluated by the Mann–Whitney *U *test. *n*, number of samples analyzed; n.s., non significant; PBMCs, peripheral blood mononuclear cells. ^a^Referred to total lymphocytes; ^b^referred to total CD4^+ ^lymphocytes.

**Figure 3:**
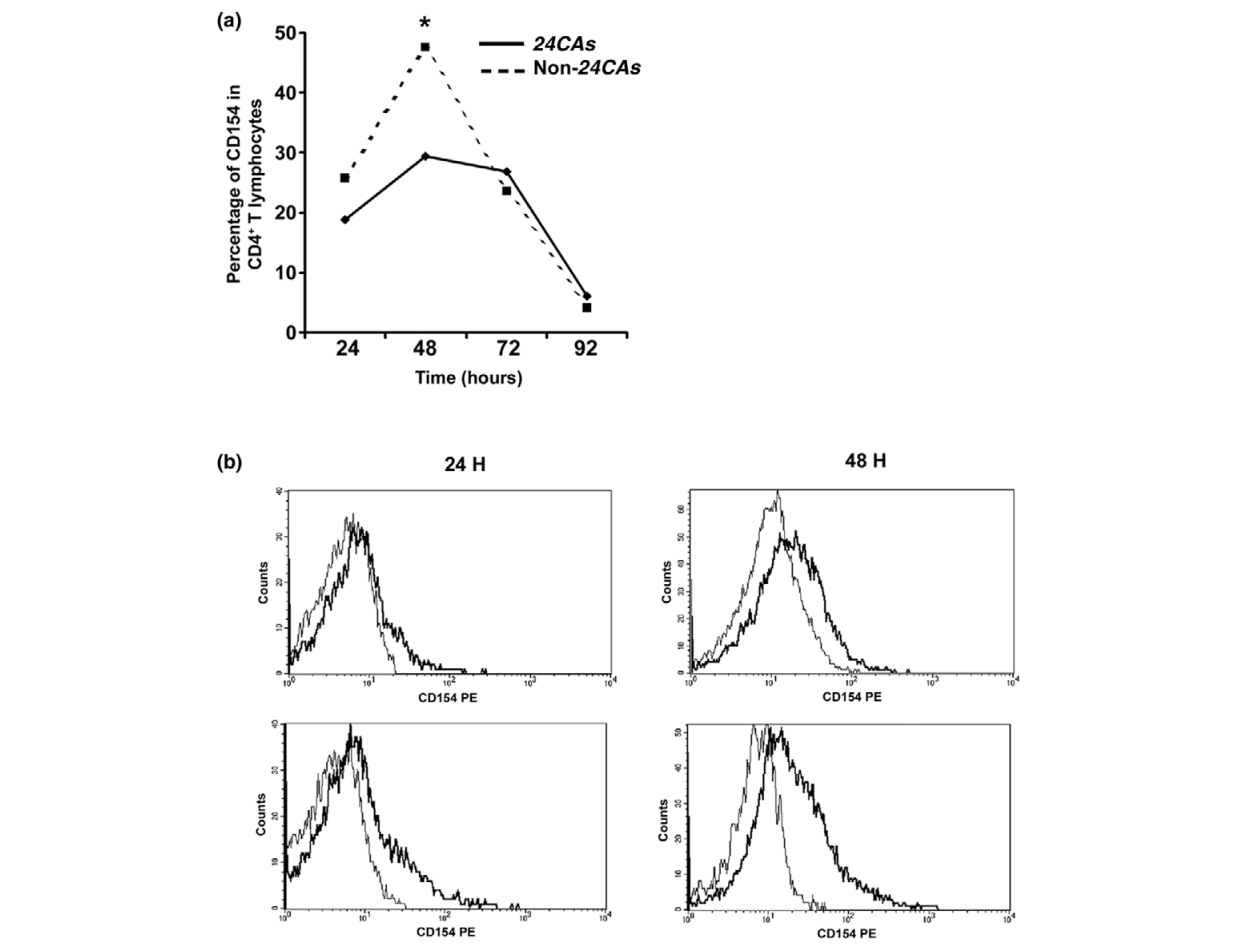
Kinetics of surface expression of CD154 in stimulated T lymphocytes **(a) **CD154 kinetic expression in CD4^+ ^T lymphocytes from patients with rheumatoid arthritis (RA) after stimulation with anti-CD3/anti-CD28, according to *CD154 *genotype. Peripheral blood mononuclear cells from patients with RA (*24CAs *group, *n *= 19; non-*24CAs *group, *n *= 16) were stimulated *in vitro *with anti-CD3/anti-CD28 for 24, 48, 72, and 92 hours. After this, the surface expression of CD154 was measured by flow cytometry. The graph shows the median percentage of CD154 expression in CD4^+ ^T lymphocytes from each group at the indicated times. *P = 0.046, comparing the *24CAs *group with the non-*24CAs *group. **(b) **Cytometry histograms showing CD154 staining (thick line) compared with non-specific staining (isotype control mAb, thin line) in CD4^+ ^T lymphocytes from representative patients with RA (upper panel, *24CAs*; lower panels, non-*24CAs*), after 24 hours (left panels) and 48 hours (right panels) of stimulation with anti-CD3/anti-CD28. PE, phycoerythrin.

To assess the influence of the microsatellite in the kinetics of surface expression of the CD154 protein, PBMCs from both groups of homozygous patients with RA were stimulated with anti-CD3 plus anti-CD28 for 24 hours or more, to allow mRNA stabilization and thus favor protein surface expression. Initially, we verified that there were no differences between groups in the numbers of apoptotic or proliferating cells, or of contaminating monocytes (data not shown), and the activation of anti-CD3-stimulated lymphocytes was confirmed in all samples by staining for CD69 and CD25 and by flow cytometry analysis (data not shown). We observed an increase in the percentage of CD154^+^CD4^+ ^lymphocytes, reaching a maximum at 48 hours in both groups, but it was lower in patients with *24CAs *alleles than in those with non-*24CAs *alleles (median 29.4% of CD154^+^CD4^+ ^lymphocytes versus 47.6%) (*P *= 0.025; Figure 3). Again, MFIs calculated as the ratio GeoMean CD154/GeoMean Ig were higher in patients with non-*24CAs *alleles but did not reach statistical significance (2.92 ± 1.09 versus 2.37 ± 0.83; *P *= 0.098).

## Discussion

In the present study we describe the association of the microsatellite in the 3' UTR of the *CD154 *gene with RA in females from the Canary Islands. The *24CAs *allele is less represented in patients than in controls, the difference being significant in women but not in men, and this gene variant protects from RA when homozygous. Different immunogenetic associations in male and female patients with RA have been described previously [42-46], although the mechanism underlying these differences is not fully understood. One possible explanation of these findings is that RA in males and females might be partly diverging disease entities, as proposed by Weyand and colleagues [47] or, alternatively, might result from the existence of differential hormonal influences. It is well known that RA is three times more frequent in women than in men, and many patients experience a clinical remission during pregnancy. However, it remains to be elucidated how hormonal differences might account for sex differences in *CD154 *association with disease susceptibility. In line with this, recent data have shown that CD154 expression could be modified by hormones such as estrogens [48] and prolactin [49].

We also demonstrate that, although *HLA-DR4 *and *HLA-DQ3 *is associated with RA in the Canary Islands population, in agreement with previous studies in Spaniards [41,44], *CD154 *association against RA is independent of HLA phenotype. These results differ from those previously obtained in Germans by Gomolka and colleagues [33], who described that the *21CAs *allele is a risk factor for patients with RA who are DR4^-^DR1^-^. Different allele frequencies between northern and southern Europe have been described for a variety of gene polymorphisms and probably contribute to the observed discrepancy, although there are other methodological reasons that also could explain the disparity of the results. Comparisons in the German population were not performed separately in men and women, and phenotype rather allele or genotype frequencies were used. Our data arranged in that way result in a similar overall distribution of phenotype frequencies to that reported by Gomolka and colleagues in both controls and patients with RA. Similarly, in our cohort 18% of DR4^-^DR1^- ^patients with RA bear a *21CAs *allele, compared with only 0.5% of DR4^- ^DR1^- ^in controls. However, we excluded the analysis of minor alleles because the small number of individuals with those alleles did not permit reliable statistical comparisons to be made.

We wished to see whether the association that we had found between the *CD154 *microsatellite and predisposition to RA was sustained by the differential expression of the gene variant associated with RA. Regulation of mRNA stability is important in controlling the expression of this gene, and the microsatellite lies close to sites of binding of protein complexes that modulate mRNA stability [29]; CA repeats have been shown to have a direct influence on mRNA stability [50], as well as on other events of gene expression [51-53]. We checked whether *24CAs *alleles displayed different behaviors in relation to mRNA decay rates and compared the *24CAs *mRNA with the other alleles. In agreement with previous studies [29,54], we confirmed that *CD154 *mRNA was more labile after 6 hours of stimulation with anti-CD3 than after 24 hours, but mRNAs with *24CAs *alleles had greater half-lives than the mRNAs from the other alleles after 6 and 24 hours of stimulation with anti-CD3.

Regarding protein expression, non-*24CAs *CD4 T cells expressed more CD154 after 48 hours of stimulation with anti-CD3/anti-CD28. Because staining for CD154 produced single-peaked histograms overlapping the negative control histograms, it is difficult to provide a precise description of CD154 distribution across CD4 T cells in terms of the percentage of positive cells and the mean MFI of the positive population. Staining of a Jurkat cell line with the same antibody resulted in two peaks, with a clear separation of CD154-positive and CD154-negative subpopulations. Dilution of the anti-CD154 antibody to mimic low CD154 expression changed the shape of the histogram to form a single peak overlaid with the control peak. In this situation, the percentages of positive cells calculated by subtracting positive and negative histograms did not reflect the true fraction of positive cells and changes according to the quantity of antibody added (data not shown). Thus, the dimly stained CD4 T cells from patients with RA in our '*in vitro*' assay seem to be reflecting a low density of CD154 on the surface, and the percentages of positive cells obtained probably do not reflect the true CD154-positive fraction of cells but still provide a rough measure of CD154 quantity in the CD4 T cell population.

Statistical analysis showed significant differences in the percentages of CD154^+ ^cells but not in the MFIs. However, data on these two results look similar and both seem to provide an expression of the higher content of CD154 in non-*24CAs *CD4 T cells. Ultimately, what is relevant is that this differential CD154 expression may be meaningful '*in vivo*', affecting the response of the immune system to autoantigens, and hence the probability of developing autoimmunity. In this regard, it is interesting that a higher CD154 protein expression in people with the non-*24CAs *allele has been consistently found in a variety of situations with similar differences between people with *24CAs *and non-*24CAs *genotypes (median percentage of CD154^+ ^cells, *24CAs*/median percentage of CD154^+ ^cells, non-*24CAs*: 0.64 in freshly isolated PBMCs, 0.62 in anti-CD3/anti-CD28 stimulated PBMCs, and 0.55 in PBMCs after 1 week of expansion with PHA/anti-CD28), supporting the idea that CD154 microsatellite alleles influence the expression of this gene after TCR engagement. Nevertheless, it is important to note that this higher CD154 expression in non-*24CAs *refers to average values, and individual percentages and MFIs substantially overlap between *24CAs *and non-*24CAs*. We lack several replications of a single individual to estimate the variation inherent in the assay, but it is likely that a significant amount of the scattering in the data is produced by interindividual variations in several genes other than CD154 that also influence the expression of this gene after T cell activation.

Results in protein expression seem to contrast with results on mRNA stability. However, there are some concerns about the comparison of mRNA and protein results because different sources of cells were used for each experiment. Direct comparison of these results should be taken with caution, because different proportions of T cell subsets could have distinct kinetic patterns of CD154 expression. Furthermore, times of stimulation in the analysis of mRNA and protein expression match at only one time point (24 hours), so these experiments cannot be paralleled.

Taking the results together, the *24CAs *allele, although conferring more stability on its mRNA, finally seems to result in a lower CD154 protein expression after activation. This means that the microsatellite alleles are associated not only with mRNA half-life, which seems plausible because of its location in the 3' UTR, but with other factors that probably affect the transcription of the gene and that result in a lower percentage of T cells expressing this protein on the surface after stimulation of the TCR. Although very speculative, this could be related to the NF-κB enhancer located near the microsatellite, which has been shown to have a crucial effect on transcription of the gene [28]. Changes in the affinity of this NF-κB site related to allelic variants of the microsatellite could lead to different thresholds for the activation of transcription, which in turn could lead to different percentages of cells expressing CD154 when stimulated.

These results therefore seem to agree with the known pattern of CD154 expression, because it has been shown that the greatest level of CD154 expression after T cell activation occurs at a time when the mRNA is being rapidly degraded and that expression is controlled by both transcriptional mechanisms and message stability [54].

More studies will be necessary to confirm the association of this microsatellite marker with RA, to establish more accurately whether the association occurs through a direct effect in the expression of the *CD154 *gene and, if so, what are the exact mechanisms by which different alleles lead to a different expression of the gene.

The present results and our previous data showing CD154 association with systemic lupus erythematosus in Canary Islanders suggest that CD154 may commonly contribute to the pathophysiological process and common immunogenetic mechanisms underlying both autoimmune diseases, thus being in agreement with the hypothesis of the 'common genetic origin' of autoimmune diseases [55,56].

## Conclusion

In the present study we report the association of the microsatellite in the 3' UTR of *CD154 *with RA in females from the Canary Islands. Differences found in mRNA decay according to *CD154 *genotypes suggest that this polymorphism may contribute to the regulation of mRNA expression, although further assays will be necessary to elucidate its role in disease predisposition. Additional studies from other series of patients will be required to confirm this genetic association.

## Abbreviations

ActD = actinomycin D; APC = antigen-presenting cell; BrdU = bromodeoxyuridine; CI = confidence interval; FITC = fluorescein isothiocyanate; HLA = human leukocyte antigen; IL = interleukin; mAb = monoclonal antibody; MFI = mean fluorescence intensity; MHC = major histocompatibility complex; NF = nuclear factor; OR = odds ratio; PBMCs = peripheral blood mononuclear cells; PCR = polymerase chain reaction; PHA = phytohemagglutinin; RA = rheumatoid arthritis; SSO = sequence-specific oligonucleotides; TCR = T-cell antigen receptor; Th = T helper type; UTR = untranslated region.

## Competing interests

The authors declare that they have no competing interests.

## Authors' contributions

TM-D and IL-F designed the study, performed the experiments, analyzed and discussed the results and prepared the manuscript. These authors contributed equally to this work, and the order of authorship is arbitrary. GP-C participated in the analysis and interpretation of the results and in manuscript preparation. IR-F, CE, and AN participated in the collection of clinical data, in the recruitment of patients and in the discussion of results. SR participated in the analysis and interpretation of the results. FS and MJC performed genotyping of the control group. AG-S participated in the collection of samples. JAV contributed to the discussion. PP-A coordinated the study, participated in its design, oversaw all aspects of the laboratory work and participated in manuscript writing and discussion. All authors read and approved the final manuscript.

## Supplementary Material

Additional file 1 An Excel containing data on the allelic frequencies of CD154 microsatellite in patients and controls. Click here for file
